# Efficiency and microbial community characteristics of strong alkali ASP flooding produced water treated by composite biofilm system

**DOI:** 10.3389/fmicb.2023.1166907

**Published:** 2023-05-25

**Authors:** Dong Wei, Xinxin Zhang, Chunying Li, Zhongting Ma, Min Zhao, Li Wei

**Affiliations:** ^1^College of Life Science, Northeast Forestry University, Harbin, Heilongjiang, China; ^2^State Key Laboratory of Urban Water Resource and Environment, Harbin Institute of Technology, Harbin, Heilongjiang, China; ^3^Guangzhou HKUST Fok Ying Tung Research Institute, Guangzhou, Guangdong, China; ^4^School of Energy and Civil Engineering, Harbin University of Commerce, Harbin, Heilongjiang, China; ^5^PetroChina Karamay Petrochemical Co., Ltd., Karamay, China

**Keywords:** produced water, moving bed biofilm reactor, microfiltration membrane, microbial community, ASP flooding

## Abstract

Strong alkali alkali-surfactant-polymer (ASP) flooding produced water is a by-product of oil recovery, and it is a stable system composed of petroleum, polyacrylamide, surfactant, and inorganic salts. Efficient, green, and safe ASP produced water treatment technology is essential for oilfield exploitation and environmental protection. In this study, an anaerobic/anoxic/moving bed biofilm reactor with a microfiltration membrane was established and assessed for the real strong alkali ASP flooding produced water (pH 10.1–10.4) treatment. The results show that the average removal rates of COD, petroleum, suspended solids, polymers and surfactants in this process are 57, 99, 66, 40, and 44%, respectively. GC-MS results show that most of the organic compounds such as alkanes and olefins in the strong alkali ASP produced water are degraded. Microfiltration membrane can significantly improve the efficiency and stability of sewage treatment system. *Paracoccus* (AN), *Synergistaceae* (ANO) and *Trichococcus* (MBBR) are the main microorganisms involved in the degradation of pollutants. This study reveals the potential and adaptability of composite biofilm system in treating the produced water of strong alkali ASP produced water.

## 1. Introduction

With the increase of industrial demand for oil and the decrease of oil reserves, the improvement of oil recovery is the focus of oil industry. Chemical flooding is an effective and economical oil recovery method (Gbadamosi et al., 2019; Arab et al., 2022), in which alkali/polymer/surfactant (ASP) flooding is an important chemical flooding oil recovery technology. ASP flooding is to extend swept volume and enhance displacement efficiency through the synergistic effect of alkali, polymer, and surfactant (Li et al., 2019). ASP flooding has been successfully applied in China Daqing Oilfield on a large scale (Yu et al., 2019; Chen et al., 2020), and has been studied and implemented in Indian and Canadian (Arab et al., 2022; Pothula et al., 2023).

Oilfield produced water is one of the largest wastewaters flows in the petroleum industry (Olajire, 2020; Samuel et al., 2022), with a significant amount being produced during petroleum production and processing. With the increase of global production activities, the output of oilfield produced water will continue to increase (Alomar et al., 2022; Amakiri et al., 2022). Oilfield produced water is a complex and changeable mixture, including organic and inorganic compounds such as oils, polycyclic aromatic hydrocarbons, organic acids, fungicides and chemicals used in fracturing operations (Al-Ghouti et al., 2019). ASP produced water contains residual oil droplets and chemicals, which has the characteristics of high emulsification, strong stability, and difficult oil-water separation (Wu D. et al., 2021; Wu M. et al., 2021), and is a kind of oilfield sewage that is difficult to treat. Because produced water contains many toxic compounds, it will do great harm to the environment and biology if it is discharged into the environment (Husain and Al-Harthi, 2023). With the increasingly strict national environmental laws and regulations and the improvement of environmental awareness of oil companies, in order to meet the environmental laws and regulations and the sustainable development of oil companies, oil companies reuse or re-inject oilfield produced water after treatment (Mao et al., 2022).

At present, petroleum oil companies often adopt physical treatment, chemical treatment and biological treatment (Ghafoori et al., 2022; Patni and Ragunathan, 2022). Physical methods are mainly used for pretreatment of removing oil and suspended solids, and chemical methods are used for removing organic pollutants in produced water. The above treatment methods have problems such as high cost and secondary pollution in the application process. Biological treatment method is a new treatment technology of oilfield sewage in recent years. Because of its low cost and environmental protection, it has become an alternative and Supplementary method for physical and chemical treatment of produced water. Biological methods to treat produced water mainly degrade and remove pollutants by microorganisms, such as conventional activated sludge (CAS) reactor, membrane bioreactor (MBR), sequencing batch reactor (SBR), moving bed biofilm reactor (MBBR), etc., are the biological treatment methods for oilfield produced water (Fulazzaky et al., 2020; Ribera-Pi et al., 2020; Lusinier et al., 2021; Nicholas and Cath, 2021). The biological treatment method also shows good ability in removing pollutants from hypersaline oilfield wastewater (Al-Malack and Al-Nowaiser, 2018; Zhang et al., 2021; Zhou et al., 2021; Cao et al., 2022).

In this study, anaerobic baffled reactor (ABR) and microporous membrane were organically combined to construct a composite bioreactor with microporous membrane, and the composite bioreactor was used to treat the strong alkaline ASP flooding produced water. By measuring the changes of oil content, suspended solids content, polymer content, surfactant content, viscosity and COD during the treatment, the application potential of composite biological system with microporous filter membrane in the treatment of produced water from alkaline ASP flooding was evaluated. In addition, GC-MS and the third generation high-throughput sequencing technology were used to study the composition of organic pollutants and functional microorganisms in the system to quantify the degradation mechanism of pollutants by functional microorganisms. The development of this work is beneficial to prove the application potential of biofilm system with microporous membrane in alkaline ASP floofing produced water.

## 2. Materials and methods

### 2.1. ASP flooding produced water and experimental setup

**FIGURE 1:**
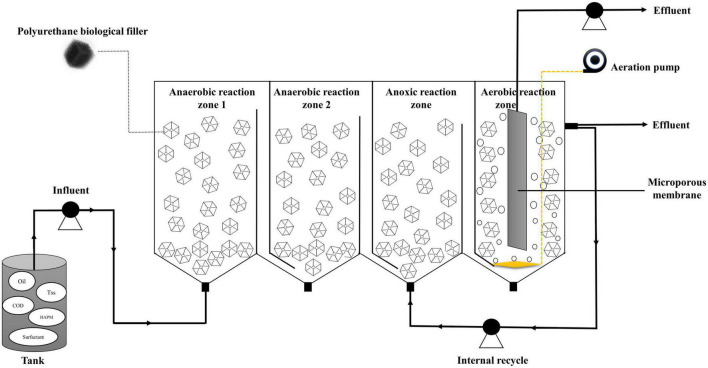
The biological experimental device.

The test sewage was taken from an ASP flooding produced water Treatment Station in Daqing Oilfield (Heilongjiang, China). The physical and chemical properties of sewage are shown in Table 1, and the produced water was alkaline because alkaline substances are used in the ASP flooding technology. Moreover, because the test wastewater was the actual production wastewater, the pollutant value fluctuates significantly. As shown in Figure 1, the anaerobic (two-stage anaerobic)/anoxic/aerobic biofilm reactor with microfiltration membrane consists of four reaction zones, with the first and second reaction zones as anaerobic reaction zones, the third reaction zone as anoxic reaction zone and the fourth reaction zone as aerobic reaction zone. The volume of each reaction zone was about 56L, and the total volume was about 224L. The microporous membrane was a flat microfiltration membrane made of polyvinylidene fluoride, which was immersed in the aerobic reaction zone. The microporous membrane was backwashed with tap water, and the membrane was cleaned with 0.5% sodium hypochlorite solution for 30 min.

**TABLE 1:** Water quality index of strong alkali ASP flooding produced water.

Parameter	Range	Parameter	Range
COD (mg/L)	2174.82∼4144.85	Surfactant content (mg/L)	190.8∼296.8
Suspended solid content (mg/L)	40.00∼206.25	Temperature (°C)	25∼35°C
Oil content (mg/L)	35.01∼2205.88	pH	10.1∼10.4
Polymer content (mg/L)	635.51∼1021.83	Viscosity (mPa⋅s)	1.59∼3.83

### 2.2. Operating conditions of the process

Each reaction zone of the composite bioreactor was filled with black polyurethane filler, with anaerobic filling ratio of 50%, anoxic filling ratio of 40% and aerobic filling ratio of 30%. Activated sludge and nutrient salt were added into the reaction zone respectively, and the activated sludge was taken from the sludge in the secondary sedimentation tank of Chengfeng Sewage Treatment Plant (Daqing, China). The experiment adopted continuous flow operation mode, and the hydraulic retention time was 72 h. During the experiment, the oxidation-reduction potential (ORP) in Anaerobic 1 reaction zone was kept at −410∼−570 mV, in Anaerobic 2 reaction zone at −550∼−650 mV, and in anoxic reaction zone at −370∼−470 mV. The aerobic reaction zone was continuously aerated by an aeration pump (SOBO SB-988, China) to ensure that the dissolved oxygen in the reaction zone was maintained at 4 ∼ 5 mg/L (Wang et al., 2019). The effluent from aerobic reaction zone flowed back to the bottom of anoxic reaction zone, and the reflux ratio was 1:1. According to the content of pollutants in influent and effluent, the test can be divided into three stages: initial start-up stage I (0 ∼ 9 days), adaptation stage II (10 ∼ 45 days) and stable operation stage III (46 ∼ 90 days).

### 2.3. Analytical procedures

#### 2.3.1. Physical and chemical analysis

The test objects were influent, effluent from anaerobic reaction zone, effluent from anoxic reaction zone, effluent from aerobic reaction zone and membrane effluent. The test items included oil content, suspended solid content, polymer content, surfactant content and viscosity. COD was determined by potassium dichromate method (Eaton et al., 2018), and Oil in sewage was measured by spectrophotometry (SY/T 0530-2011). Gravity method (SY/T 0530-2011) was used to measure the suspended solids content of sewage, and the measuring equipment was JBFT-03 suspended solids analyzer (Harbin Jingbo Technology Development Co., Ltd., China). The polymer content was measured by turbidity method (Q/SY DQ0928—2011), and the surfactant was measured by direct two-phase titration (GB/T 5173-2018). The viscosity of sewage was tested by rheometer (AR1500ex, US), and the testing method was rotation method. To master the operation characteristics of each reaction zone, WTW Multi 3420 multi-parameter water quality analyzer (WTW, Germany) was used to monitor the temperature, pH value and ORP in each reaction zone. There were many kinds of residual organic pollutants in alkaline ASP flooding produced water, mainly petroleum substances, residual polymers, surfactants, etc. The main purpose of the study was to degrade these organic pollutants. Residual organic substances in alkaline ASP flooding produced water mainly include crude oil, polymers, and surfactants, which leads to high polymer content, small oil droplet size, strong emulsification, and high viscosity of sewage (Li et al., 2021). Petroleum pollutants and polymers will pose a serious threat to the environment if they are not treated effectively (Song et al., 2019a,^2021^; Patni and Ragunathan, 2022). In order to quantify the removal of organic pollutants such as petroleum substances and polymers by bioreactor in this study, during the stable operation of the reactor, the influent, effluent from anaerobic reaction zone 1, effluent from anaerobic reaction zone 2, effluent from anoxic reaction zone, effluent from aerobic reaction zone and membrane effluent were sampled, and then the organic pollutants in the samples were measured by GC-MS. 7890A GC-5975C MS (Agilent, US) was used to measure the composition of organic pollutants. Methyl tert-butyl ether and methyl chloride were used as extractant, high-purity N_2_ (99.999%) was used as carrier gas, and H_2_ and air were used as fuel gas. When the ion source temperature reached 240°C, the detection began, and the peak spectra of the results were compared and analyzed to obtain the composition and distribution of organic pollutants in the samples (Qian et al., 2022).

#### 2.3.2. Microbial community analysis

High-throughput sequencing was used to analyze the biofilm on the packing of anaerobic reaction zone 1 (AN1), anaerobic reaction zone 2 (AN2), anoxic reaction zone (ANO) and aerobic reaction zone (O) in the stable period, to obtain the microbial community characteristics involved in the degradation of pollutants in wastewater. DNA of microbial samples was extracted by E.Z.N.A.^®^ Soil DNA Kit (Omega Bio-tek, Norcross, GA, USA), and 338f (5′- ACTCCTACGGGAGGCAGCAG -3′) and 806r (5′- GGACTACHVGGGTWTCTAAT) were measured by ABI GeneAmp^®^ 9700 PCR Thermocycler (ABI, CA, USA). The PCR mixtures contain 5 × *TransStart* FastPfu buffer 4 μL, 2.5 mM dNTPs 2 μL, forward primer (5 μM) 0.8 μL, reverse primer (5 μM) 0.8 μL, *TransStart* FastPfu DNA Polymerase 0.4 μL, template DNA 10 ng, and finally ddH_2_O up to 20 μL. PCR reactions were performed in triplicate. The PCR product was extracted from 2% agarose gel and purified using the AxyPrep DNA Gel Extraction Kit (Axygen Biosciences, Union City, CA, USA) according to manufacturer’s instructions and quantified using Quantus Fluorometer (Promega, USA).

In this study, the purified amplicons were combined in equal amounts and subjected to paired-end sequencing using either an Illumina MiSeq PE300 platform (Illumina, San Diego, USA). The sequencing process followed the standard protocols established by Majorbio Bio-Pharm Technology Co. Ltd. (Shanghai, China) (Ren et al., 2022). The original sequence of MiSeq sequencing was assembled and filtered to remove low-quality reads, and then it was clustered into ninety-seven percent-like operational taxa, OTU) by Markov clustering algorithm. The water abundance table and beta diversity distance of each taxonomy were calculated by QIIME program,^1^ and the α diversity index (Chao, Simpson and Shannon) was calculated by Mothur program.^2^ In the process of BLAST search, according to the comparison between 16S rRNA sequence and nucleotide sequence database, the closest genetic relationship of OTU was determined. Microbiological analysis was carried out by using Silva^3^, and NCBI^4^ databases.

## 3. Results and discussion

### 3.1. Reactor performance

Because there are a lot of alkaline substances and surfactants in sewage, the interface of strong alkali ASP flooding produced water is more stable than that of polymer-containing sewage, and it is more difficult to treat. Therefore, the performance of composite bioreactor was evaluated by the removal rates of petroleum substances, suspended solids, polymers, surfactants, viscosity, and COD. The content of petroleum substances and suspended solids were used as conventional pollution indicators of produced water, and the average removal rates of petroleum substances and suspended solids were 98.31% and 64.02% respectively. Zhang et al. (2019) prepared Aluminum and iron leaching from power plant coal fly ash for preparation of polymeric aluminum ferric chloride flocculant from fly ash, and the removal rate of petroleum in polymer-containing wastewater reached 91.5% respectively. Compared with flocculation process, this system has a better removal effect on petroleum substances, and it does not need to add chemical flocculant, which can reduce the treatment cost. Polymer and surfactant were typical pollutants in ASP flooding produced water, and the average removal rates of polymer and surfactant in the whole test period were 37.51% and 44.36% respectively. In addition, the biological action of microorganisms on polymers destroyed the original stable system of sewage, and the viscosity of sewage decreased by 38.92%. As an index of organic pollutant removal efficiency, the average removal rate of COD was 57.14%. Composite bioreactor can significantly remove the content of oily substances in wastewater and show degradation characteristics to typical pollutants such as polyacrylamide and surfactant and can destroy the stability of produced water and reduce the viscosity of produced water.

#### 3.1.1. Oil and suspended solids removal

**FIGURE 2:**
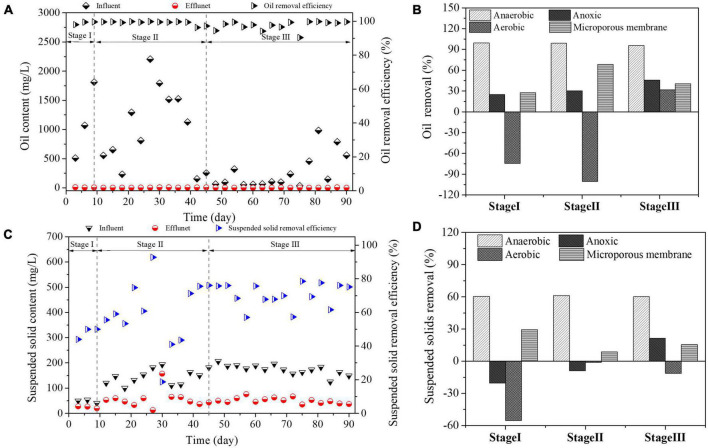
Changes of petroleum concentration and suspended solid concentration during the test. **(A)** Change of oil content concentration in inlet and outlet water; **(B)** removal of oil content in each reaction zone; **(C)** change of suspended solid content concentration in inlet and outlet water; **(D)** removal of suspended solid content in each reaction zone.

Oil content and suspended solids content are important indexes to evaluate the treatment effect of produced water. The oil content removal effect of the reactor is shown in Figures 2A, B. Because the test sewage was produced during the actual operation on site, the test sewage fluctuates. During the whole test, the oil content of the influent was in the range of 652.811 ± 617.15 mg/L, and the oil content of the effluent at different stages was 7.44 ± 1.98 (Stage I), 4.35 ± 2.29 (Stage II) and 2.53 ± 1.66 (Stage III) respectively. During the test period, most of the oil removal rate remained above 98%, and microorganisms could metabolize petroleum substances in wastewater to degrade petroleum substances in wastewater (Purnima et al., 2023). The oil content removal rate of each reaction zone is shown in Figure 2B. The anaerobic reaction zone is the main unit for oil content removal, with the removal rates of 99.31% (Stage I), 98.96% (Stage II) and 95.76% (Stage III) respectively. Previous studies have shown that biological contact oxidation can significantly reduce petroleum substances and organic pollutants in sewage in the actual treatment of oilfield produced water (Zhou et al., 2021). In this study, the degradation of petroleum substances mainly occurred in the anaerobic process section. Under anaerobic conditions, long-chain petroleum substances were converted into small molecular substances through hydrolysis and acidification. Surprisingly, the oil content in the aerobic reaction zone increased, which was due to the microbial metabolic activity and aeration destroying the stability of the hydration membrane of some emulsified oil (O/W) in water, releasing oil-containing substances inside, leading to the increase of oil content. With the experiment, the removal rate of oil content in anoxic reaction zone increased from 24.92 (Stage I) to 45.93% (Stage II), and the increase of oil content in effluent of aerobic reaction zone disappeared, and the removal rate of oil content was 31.62% (Stage III), indicating that microorganisms in anoxic reaction zone enhanced the destruction of oil-in-water type, released emulsified oil, reduced the release of emulsified oil in aerobic section and increased the aerobic reaction zone. In addition, the microporous membrane can effectively reduce the content of petroleum substances in biological effluent during the whole test period and ensure the treatment efficiency of bioreactor.

The suspended solids content removal effect of the reactor is shown in Figures 2C, D. Suspended solids formed by the precipitation of tiny mineral particles in alkaline ASP flooding produced water can increase the stability of the produced water emulsion and increase the difficulty of treating the produced water (Wu D. et al., 2021). The content of suspended solids in influent was 40.00∼206.25 mg/L, and the content of suspended solids in effluent was 24.89 ± 3.50 (Stage I), 56.68 ± 33.53 (Stage II) and 51.20 ± 11.26 (Stage III) during the whole test period, which shows that the system can significantly reduce suspended solids in wastewater. During the growth of biofilm, the membrane will fall off, resulting in the increase of total suspended solids in effluent (Andreottola et al., 2000). Suspended solids in anoxic reaction zone and aerobic reaction zone increased during the initial start-up period and microbial adaptation period of the reactor. With the growth of biofilm becoming stable, biofilm shedding in anoxic zone eased, and the treatment efficiency of suspended solids gradually increased from −20.33% (Stage I) to 21.40% (Stage III). However, due to aeration, the filler in the aerobic reaction zone was in a dynamic state, and the biofilm was in a dynamic balance of growth and leakage, which always showed the phenomenon of suspended solids rising. High suspended solids content can easily lead to formation plugging and other problems in the process of produced water reinjection. The content of suspended solids after filtration by microporous membrane is stable at about 50mg/L, which indicates that microporous membrane can effectively maintain the removal efficiency of suspended solids in the reactor.

#### 3.1.2. Removal efficiency of polymers and surfactants

**FIGURE 3:**
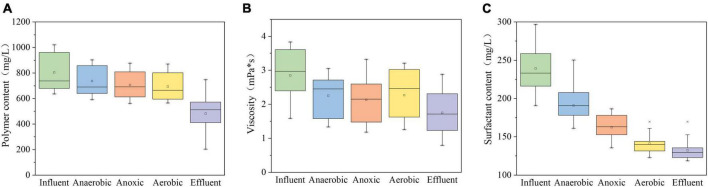
Changes of typical pollutants in strong alkali ASP flooding produced water. **(A)** Change of polymer content; **(B)** viscosity change of sewage; **(C)** change of surfactant content.

In previous studies on the influence of alkalinity, surfactants, and polymers on the emulsification of ASP flooding produced fluid, it was found that surfactants reduced the interfacial tension between oil and water under alkaline conditions (Bashir et al., 2022), and the use of polymers provided the viscosity of water phase (Afolabi et al., 2022), which led to the enhancement of O/W emulsification of produced fluid (Pashapouryeganeh et al., 2022). It is possible for polyacrylamide (HPAM) to produce acrylamide (AM). Previous studies have shown that AM has carcinogenicity and reproductive toxicity (Pundir et al., 2019). Therefore, the removal of polymers and surfactants is particularly important in the treatment of alkaline ASP produced water. Hydrolytic polyacrylamide (HPAM) has the characteristics of large molecular weight (Li et al., 2019), and the wastewater is alkaline (pH = 10.1∼10.4), which further increases the difficulty of microbial degradation. The content of polymer in the influent was 804.94 ± 139.18 mg/L during the whole test period. It can be seen from Figure 3A that the anaerobic reaction zone (737.43 ± 109.56 mg/L), the anoxic reaction zone (706.29 ± 97.42 mg/L) and the aerobic reaction zone (693.67 ± 101.53 mg/L) all have certain degradation ability to polymer content. However, the average removal rate of polymer content in the whole biological stage is 13.82%, and the removal effect is not good, because the polymer has a stable structure, which reduces the ability of microbial organisms to degrade it. As a supplementary means of composite biological system, microporous membrane can obviously reduce the polymer content in wastewater. The polymer content in the effluent of microporous membrane was 482.62 130.47 mg/L, and the average removal rate of pollutants was 40.04%. Polymer in ASP flooding technology improves water phase viscosity, reduces effective water phase permeability and oil-water mixed phase fluidity, thus improving oil recovery (Li et al., 2019). Therefore, the viscosity of produced water is the main index to evaluate the stability of produced water. Figure 3B shows that the composite bioreactor can reduce the viscosity of wastewater, from 2.85 ± 0.75 mPa⋅s in influent to 1.75 ± 0.64 mPa⋅s in effluent, with an average viscosity reduction rate of 38.59%, and the stability in wastewater is reduced. Combined with the change of polymer content in Figure 3A, it is inferred that although microorganisms cannot significantly reduce the polymer content, they will destroy the structural stability of polymer, leading to instability of wastewater and reduce the viscosity in wastewater. Surfactants can significantly change the internal interfacial tension of sewage and play a role in maintaining the stability of the whole sewage. As can be seen from Figure 3C, the influent content of surfactant is 239.28 ± 28.92 mg/L, which shows gradient degradation in the composite bioreactor. The average removal rates in anaerobic, anoxic, aerobic, and microporous membranes were 20.20%, 14.94%, 13.05% and 6.31% respectively, and the average effluent concentration was 132.29 ± 12.16 mg. Therefore, the composite bioreactor can effectively degrade polymers and surfactants in wastewater under alkaline conditions and destroy the stability of sewage. Song et al. (2019b) found that *Bacillus megaterium strain* SZK-5 could degrade polymer in the indoor experiment, and the removal rate was 55.93%. Based on the complexity of strong alkali ASP flooding produced water, the average removal rate of polymer in the actual treatment process of this system reached 40.04%, and the viscosity of sewage decreased by 38.59%, which showed that the composite bioreactor could effectively degrade polymers and surfactants in wastewater under alkaline conditions and destroy the stability of sewage.

#### 3.1.3. COD removal

**FIGURE 4:**
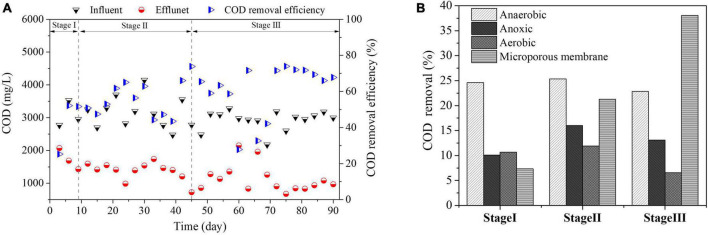
Changes of COD content and removal rate. **(A)** Changes of COD content in influent and effluent; **(B)** COD removal rate in each reaction zone.

The changes of COD content and removal rate in influent and effluent of bioreactor are shown in Figure 4. Due to the fluctuation of water quality, the influent COD concentration was 2174.82 ∼ 4144.85 mg/L. At the initial stage of start-up, the COD content of effluent was 1728.67 ± 263.63. With the continuous adaptation of microorganisms to pollutants, the COD removal efficiency increased, and the COD content of effluent was 1368.83 ± 266.21 (Stage II). When the reactor entered the stable operation period, the effluent COD was 1137.40 ± 406.06, and the average removal rate was 60.87%. Biofilm method can effectively reduce petroleum substances in produced water (Zhou et al., 2021). Fulazzaky et al. (2020) tried to treat the produced water of simulated oil fields by submerged MBR. The experiment showed that the removal efficiency of COD and grease by MBR system could reach over 90%. In this study, the removal rate of COD in the composite bioreactor is lower than that of MBR membrane process, but the average removal rate of petroleum substances in the composite bioreactor is as high as 98%. Considering the types of pollutants (petroleum substances, polymers, surfactants, etc.) in the strong alkali ASP flooding produced water, it can be seen that polymer is not easily degraded by microorganisms, resulting in a low removal rate of COD.

**FIGURE 5:**
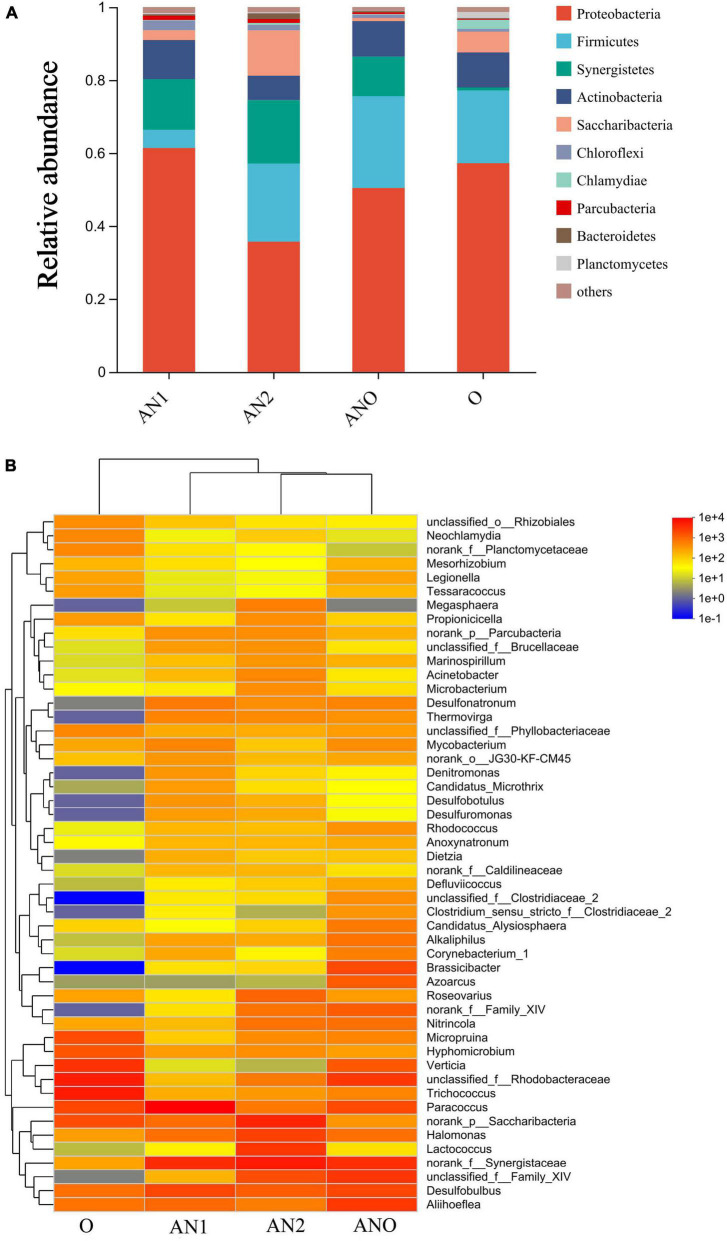
Distribution of bacteria in the composite bioreactor. **(A)** Histogram of bacterial composition at the phylum level; **(B)** heatmap of bacterial composition at genus level.

*Synergistaceae* occupies a prominent position in the anaerobic reaction zone in the composition of bacteria shown in Figure 5, which can convert hydrocarbons into small molecules during anaerobic process. According to the oil content removal situation (see Section “3.1.1. Oil and suspended solids removal” for detail), the removal of COD in wastewater mainly comes from the degradation of petroleum substances in sewage. As shown in Figure 4B, the removal rates of COD in each reaction zone are 24.59, 10.10, 10.69, and 7.36% respectively in the initial stage of start-up. As the main unit of COD removal in the initial stage of the reactor, the removal rate of anaerobic reaction zone did not increase significantly with the extension of operation time, which was 25.35% (Stage II) and 22.83% (Stage III) respectively. It shows that petroleum substances in alkaline ASP produced water are almost biodegraded and transformed in the anaerobic stage, and some surfactants are degraded and transformed (Figure 3C). The effect of anaerobic on polymer is manifested in the destruction of polymer structural stability by its own metabolic activity, which leads to the decrease of viscosity in wastewater (Figure 3B). The removal rate of COD in the anoxic reaction zone were 16.02% (Stage II) and 13.09% (Stage III), which was higher than that in the start-up stage. Due to the enhanced adaptability of microorganisms to pollutants and environmental factors (ORP: −370 ∼−470 mV) after domestication, anoxic microorganisms such as *Aliihoeflea*, *Rhodobacteraceae*, *Brassicibacter* become functional microorganisms. The removal rate of COD in aerobic reaction zone was 6.55% (Stage III) in stable operation stage, which was the process of biofilm renewal and shedding all the time during MBBR operation. The concentration of organic suspended solids in wastewater increased, resulting in high concentration of organic matter in effluent. The microporous membrane can intercept the biofilm shed by MBBR, and the removal rate of COD by microporous membrane is 21.28% (Stage II) and 38.05% (Stage III) respectively, which shows that microporous membrane can effectively solve the problem of COD increase in aerobic effluent.

### 3.2. Organics biodegradation

**FIGURE 6:**
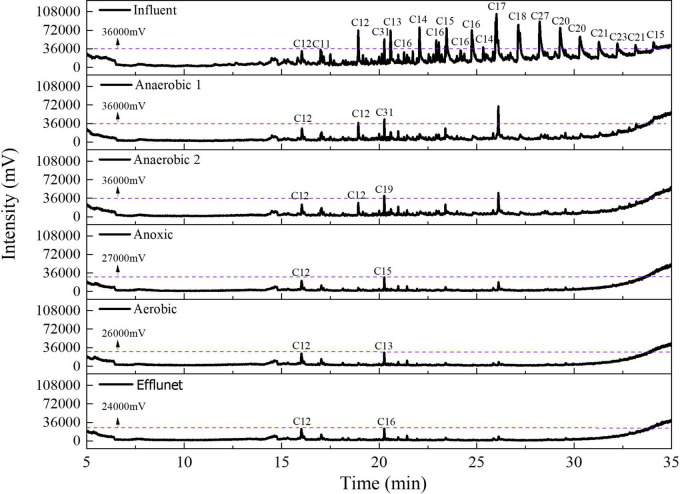
GC-MS spectrum of organic pollutants. Cn represents the number of carbon atoms in the compound.

To quantify the degradation of organic pollutants in anaerobic reaction zone, anoxic reaction zone, aerobic reaction zone and microporous membrane, GC-MS was used to analyze the composition of organic substances in stable samples, and the results are shown in Figure 6. There are more than 50 kinds of organic substances in the influent, and the peak intensity is used as the evaluation index, and the organic pollutants with the peak intensity greater than or close to 36000mV are selected. Decane, 3,6- dimethyl-, Undecane, Dodecane, Nonane,3- methyl-, Hentriacontane, Tetradecane, 2- cyclohexyl-, Pentadecane, Hexadecane, Decane,2- cyclohexyl-, Bicyclo[3.1.1]heptan-2-one,6,6- dimethyl-, (1R)-, Tridecane,3- methyl-, Octadecane, Eicosane, Sulfurous acid, cyclohexylmethyl octadecyl ester, Tricosane, 2-Methyl-7-phenylindoleand so on are the main pollutants in the influent. After two-stage anaerobic treatment, there are about 27 kinds of organic pollutants in sewage, but only sulfurous acid, octadecyl 2-propyl ester and Nonadecane are greater than or close to 36000mV. There are 10 kinds of organic pollutants in the effluent of anoxic reaction zone, but their peak intensities are all less than 36000mV, among which Pentadecane has the largest peak intensity (27593 mV). There are 11 kinds of organic pollutants in the effluent of aerobic reactor, among which Decane,2,3,7-trimethyl- has the highest peak intensity (26535 mV). There are 6 kinds of organics in the effluent of microporous membrane, among which Hexadecane has the highest strength (24049 mV). Therefore, the composite bioreactor can effectively degrade organic pollutants in alkaline ASP flooding produced water.

According to the distribution of organic pollutants (Supplementary Table 1), there are many kinds of organic pollutants in the influent, which are greatly reduced after two-stage anaerobic treatment, and more than 30 kinds of new organic pollutants appear. Among them, there are more than 20 kinds of Tetradecane, 4-ethyl- etc., in anaerobic reaction zone 1, and more than 10 kinds of 2′, 4′-dihydroacetophenone, bis (Trimethylsilyl) ether etc., in anaerobic reaction zone 2. Previous studies have found that anaerobic microorganisms can transform macromolecular organics in produced water into micromolecule organics by hydrolytic fermentation, which can be used for further degradation of pollutants by subsequent anoxia and good technology (Zhang et al., 2021). After anaerobic treatment, the number of carbon atoms of pollutants decreased, indicating that two-stage anaerobic treatment can effectively transform long-chain organic pollutants into small molecular pollutants. After anoxic treatment, the types of pollutants in sewage are reduced (from 27 to 10), and there are organic substances in the effluent, such as Decane, 2,3,5,8- tetramethyl-, Oxalic acid, Cyclohexylmethyl Undecylester, Methane, tricyclohexyl- and so on. This is due to the high diversity of microorganisms in the anoxic reaction zone, which can utilize pollutants that anaerobic microorganisms cannot use to realize the gradient degradation of pollutants. After aerobic treatment, there are 11 kinds of organic substances in sewage, among which Decane,3,7- dimethyl-, 10-Methylnonadecane, Dodecane, 3-methyl- and other organic substances are added to the effluent of aerobic reaction zone. This is because the degradation mechanism of aerobic microorganisms is different from anaerobic and anoxic microorganisms. Under aerobic conditions, microorganisms grow and metabolize organic substances such as residual hydrocarbons in sewage as nutrients. Due to different metabolic modes, aerobic microorganisms further degrade long-chain pollutants into new short-chain pollutants, resulting in an increase in the types of pollutants. At the same time, the aeration process destroys the emulsification of wastewater, and the wrapped pollutants are released, which increases the types of pollutants in wastewater. After membrane filtration, the types of pollutants were further reduced from 11 to 7, and the peak intensity of organic matter in the effluent from microporous filtration was less than 25000mV. This shows that the degradation mode of pollutants in alkaline ASP flooding produced water by composite bioreactor is gradient degradation, and pollutants are degraded step by step by functional microorganisms in different reaction zones (Figure 5), and microporous membrane is the most effective means to ensure pollutants and deeply remove pollutants.

### 3.3. Microbial community analysis

#### 3.3.1. Microbial diversity

As can be seen from Table 2, the available sequence numbers of anaerobic reaction zone 1, anaerobic reaction zone 2, anoxic reaction zone and aerobic reaction zone are 41,562, 45,689, 48,998 and 39,580 respectively. The effective sequences in two anaerobic reaction zones formed 221 and 146 OTU units respectively, 48,998 sequences in anoxic reaction zone formed 104 OTU units, and 39,580 sequences in aerobic reaction zone formed 201 OTU units. The coverage rates of microbial libraries of the four samples were all over 99.9%, which indicated that the sequencing results basically covered all the microbial biomass in the samples, and the measurement results could truly reflect the true value of the samples (Zhang et al., 2018). Shannon and Simpson were used to evaluate the diversity of samples. The greater Simpson index value, the lower community diversity, and the greater Shannon value, the higher community diversity. The order of AN1, AN2, ANO and O diversity was ANO > AN2 > O > AN1. The distribution of diversity shows that when AN1 is impacted by strong alkali ASP flooding produced water, the functional microbial population increases sharply, and the diversity decreases significantly to ensure the continuous adaptation of microorganisms to pollutants in wastewater. As shown in Supplementary Figure 1A, it is the PCoA analysis of different process samples. It can be seen from the figure that the distance between AN2 and ANO sample points is relatively close, indicating that the microbial composition of the two samples is highly similar. The distance between AN1 and O samples is far away, indicating that the sample composition is different, which is related to the distribution of functional areas in biotechnology. As shown in Supplementary Figure 1B, it is the hierarchical cluster analysis of samples OTU. From the length of branches in the figure, it can be seen that the distance between AN2 and ANO samples is relatively close, and the O sample is significantly different from other samples, because the dissolved oxygen concentration in aerobic process environment is significantly higher than that in anoxic anaerobic environment.

**TABLE 2:** Biodiversity index of microbial communities in samples.

Sample ID	Reads	OTU	Chao	Cover- age	Shannon	Simpson
AN1	41562	221	1132	0.999490	3.85	0.1232
AN2	45689	146	1029	0.999798	4.40	0.0346
ANO	48998	104	870	0.999245	4.26	0.0342
O	39580	201	1095	0.999355	3.58	0.0811

#### 3.3.2. Composition and distribution of microorganisms

As shown in Figure 5A, it is the distribution of microbial community composition at the phylum level. The bacteria in anaerobic reaction zone 1, anaerobic reaction zone 2 and anoxic reaction zone mainly including *Proteobacteria*, *Firmicutes* and *Synergists*. The microorganisms in aerobic reaction zone are mainly *Proteobacteria*, *Firmicutes*, *Actinobacteria* and *Saccharibacteria*. *Proteobacteria* are the dominant microorganisms in the three reaction zones, and *Proteobacteria* bacteria play an important role in the process of petroleum degradation (Li et al., 2022; Xiao et al., 2022).

The distribution of microorganisms at the genus level is shown in Figure 5B. *Paracoccus* (33.0%), *norank f Synergistaceae* (12.0%), *Desulfobulbus* (6.4%), *Aliihoeflea* (2.97%), *norank p Saccharibacteria* (2.7%) and *Halomonas* (2.5%) have relative abundance greater than 2% in anaerobic reaction zone 1. *Paracoccus* can increase its ability to remove petroleum hydrocarbons by producing biosurfactants (Xu et al., 2020). Previous studies have found that *Desulfobulbus* can participate in the biodegradation of polycyclic aromatic hydrocarbons (Bai et al., 2021). *Paracoccus* is a functional microorganism for oil degradation in anaerobic reaction zone 1. Main microbial composition of *Synergistaceae* in anaerobic reaction zone 2. *Synergistaceae* has the ability to degrade hydrocarbons and can participate in the hydrocarbon degradation process of pollutants (Sierra-Garcia et al., 2020). *Synergistaceae* in anaerobic reaction zone 2 mainly degrades small molecules containing carbon-hydrogen bonds. The anoxic reactor mainly includes the genera *Synergistaceae* (9.7%), *Aliihoeflea* (7.6%), *Rhodobacteraceae* (7.5%), *Desulfobulbus* (5.3%), *Brassicibacter* (5.0%). The composition ratio of various bacteria is similar, which can coordinate with each other and improve the removal ability of complex pollutants, among which *Rhodobacteraceae* is a facultative anaerobic bacterium with the ability to degrade hydrocarbon substances (Gao et al., 2022). The aerobic reaction zone mainly includes: *Trichococcus*(18.5%), *Rhodobateraceae* (16.9%), *Verticia* (11.1%), *Paracocci* (6.7%), *Saccharibactria* (5.7%) and *Micropruina* (5.7%), among which *Trichococcus* participates in the degradation process of polymer as a functional bacterium (Song et al., 2018).

## 4. Conclusion

The composite biofilm reactor with microporous filter membrane was used to treat strong alkaline ASP flooding produced water, and the pollutant treatment capacity and biodegradation mechanism in different reaction zones were evaluated. The results show that under the conditions of high pH, high emulsification and high COD, the composite biofilm reactor with microporous membrane can effectively remove pollutants from the produced water of ASP flooding with strong alkali, and the microporous membrane can improve the sewage treatment efficiency, in which the oil content of the effluent is less than 5mg/L during stable operation, which meets the standards of oilfield produced water reinjection. The identification of organic pollutants by GC-MS found that most organic pollutants were degraded and transformed. *Paracoccus* and *Synergistaceae* are involved in the degradation of pollutants such as petroleum substances and surfactants, and *Trichococcus* is involved in the degradation of polymers. In this study, a feasible composite bioreactor was established to treat the strong alkaline ASP flooding produced water, and the system can form a combined treatment process with the existing sedimentation-filtration process as the core. In case of complex contamination, toxicology assays are needed to show that the toxicity of the target contaminant is reduced after biological treatment, especially if some new peaks are observed after some stages, it may show that biotransformation is part of the process, so further studies are needed to show that toxicity of substances in the final effluent.

## Data availability statement

The original contributions presented in this study are included in the article/Supplementary material, further inquiries can be directed to the corresponding authors.

## Author contributions

DW: writing – original draft and conceptualization. XZ: data curation and writing – review and editing. CL: project administration. ZM: investigation. MZ: supervision. LW: funding acquisition and resources. All authors contributed to the article and approved the submitted version.

## Footnotes

1
http://qiime.org/install/index.html

2
https://www.mothur.org/wiki/Download_mothur

3
http://www.arb-silva.de/

4
http://ncbi.nlm.nih.gov/
