# Supplementary material for: Efficiency and microbial community characteristics of strong alkali ASP flooding produced water treated by composite biofilm system
Source: Front Microbiol. 2023 May 25;14:1166907. doi: 10.3389/fmicb.2023.1166907 (PMC10247963; doi:10.3389/fmicb.2023.1166907)
Supplement: Supplementary file 1 [file Data_Sheet_1.docx]

Supplementary Material

Article Title Efficiency and microbial community characteristics of strong alkali ASP flooding produced water treated by composite biofilm system

Dong Wei^1^, Xinxin Zhang ^2,3^, Chunying Li ^4^, Zhongting Ma^5^, Min Zhao^1^*, Li Wei^2,3^*

*** Correspondence:** Min Zhao: 82191513@163.com; Li Wei: weilihit@126.com

# Supplementary Figures and Tables

Table S1 Distribution of organic matter in each reaction zone

| Organic compound | Influent | AN1 | AN2 | ANO | O | Efflunet |
| --- | --- | --- | --- | --- | --- | --- |
| 1,1'-Bicyclopentyl | **+** | **-** | **-** | **-** | **-** | **-** |
| Decane, 3,6-dimethyl- | **+** | **-** | **+** | **-** | **-** | **+** |
| Tetradecane, 2,6,10-trimethyl- | **+** | **-** | **-** | **-** | **-** | **-** |
| Undecane | **+** | **+** | **+** | **-** | **-** | **-** |
| trans-Decalin, 2-methyl- | **+** | **-** | **-** | **-** | **-** | **-** |
| 1-Methyldecahydronaphthalene | **+** | **-** | **-** | **-** | **-** | **-** |
| Cyclohexane, pentyl- | **+** | **-** | **-** | **-** | **-** | **-** |
| Cyclopentasiloxane, decamethyl- | **+** | **+** | **+** | **-** | **-** | **-** |
| Dodecane, 2,6,10-trimethyl- | **+** | **-** | **-** | **-** | **-** | **-** |
| Carbonic acid, dodecyl isobutyl ester | **+** | **-** | **-** | **-** | **-** | **-** |
| trans,trans-1,6-Dimethylspiro[4.5]decane | **+** | **-** | **-** | **-** | **-** | **-** |
| 6,10,13-Trimethyltetradecanol | **+** | **-** | **-** | **-** | **-** | **-** |
| Dodecane | **+** | **+** | **+** | **+** | **-** | **-** |
| Undecane, 2,6-dimethyl- | **+** | **-** | **-** | **-** | **-** | **-** |
| Cyclohexane, 2-butyl-1,1,3-trimethyl- | **+** | **-** | **-** | **-** | **-** | **-** |
| cis, cis-3-Ethylbicyclo[4.4.0]decane | **+** | **-** | **-** | **-** | **-** | **-** |
| Cyclohexane, (2-methylpropyl)- | **+** | **-** | **-** | **-** | **-** | **-** |
| 2-Tetradecanol | **+** | **-** | **-** | **-** | **-** | **-** |
| Sulfurous acid, 2-propyl undecyl ester | **+** | **+** | **-** | **-** | **-** | **-** |
| Nonane, 3-methyl- | **+** | **-** | **-** | **-** | **-** | **-** |
| Hentriacontane | **+** | **+** | **-** | **-** | **+** | **-** |
| Tridecane | **+** | **+** | **+** | **-** | **-** | **-** |
| Sulfurous acid, nonyl 2-propyl ester | **+** | **-** | **-** | **-** | **-** | **-** |
| Decane, 2-cyclohexyl- | **+** | **-** | **-** | **-** | **-** | **-** |
| Cyclohexanone, 3-butyl- | **+** | **-** | **-** | **-** | **-** | **-** |
| Cyclohexane, 1-(cyclohexylmethyl)-4-methyl-, cis- | **+** | **-** | **-** | **-** | **-** | **-** |
| Dodecane, 2,6,10-trimethyl- | **+** | **-** | **-** | **-** | **-** | **-** |
| 2-Dodecanol | **+** | **-** | **-** | **-** | **-** | **-** |
| Tetradecane | **+** | **-** | **-** | **-** | **-** | **-** |
| Spiro[5.6]dodecane-1,7-dione | **+** | **-** | **-** | **-** | **-** | **-** |
| trans, cis-3-Ethylbicyclo[4.4.0]decane | **+** | **-** | **-** | **-** | **-** | **-** |
| Dodecane, 2-cyclohexyl- | **+** | **-** | **-** | **-** | **-** | **-** |
| Hexadecane | **+** | **-** | **-** | **-** | **-** | **+** |
| Decahydro-4,4,8,9,10-pentamethylnaphthalene | **+** | **+** | **+** | **-** | **-** | **-** |
| trans-anti-trans-Tetra-decahydroanthracene | **+** | **-** | **-** | **-** | **-** | **-** |
| Octadecane, 3-ethyl-5-(2-ethylbutyl)- | **+** | **+** | **-** | **+** | **+** | **-** |
| Pentadecane | **+** | **-** | **-** | **+** | **-** | **-** |
| Bicyclo[3.1.1]heptan-2-one, 6,6-dimethyl-, (1R)- | **+** | **-** | **-** | **-** | **-** | **-** |
| Tetrapentacontane, 1,54-dibromo- | **+** | **-** | **-** | **-** | **-** | **-** |
| 3,7-Dimethyl-6-nonen-1-ol acetate | **+** | **-** | **-** | **-** | **-** | **-** |
| Hexadecane | **+** | **-** | **-** | **-** | **-** | **-** |
| Tridecane, 3-methyl- | **+** | **-** | **-** | **-** | **-** | **-** |
| Undecane, 3-cyclohexyl- | **+** | **-** | **-** | **-** | **-** | **-** |
| 1H-Pyrazole-1-carboxaldehyde, 4-ethyl-4,5-dihydro-5-propyl- | **+** | **+** | **-** | **-** | **-** | **-** |
| Heptadecane | **+** | **-** | **-** | **-** | **-** | **-** |
| Tridecane, 4-cyclohexyl- | **+** | **-** | **-** | **-** | **-** | **-** |
| Octadecane | **+** | **-** | **-** | **-** | **-** | **-** |
| 2-Piperidinone, N-[4-bromo-n-butyl]- | **+** | **-** | **-** | **-** | **-** | **-** |
| Heptacosane | **+** | **-** | **-** | **-** | **-** | **-** |
| Eicosane | **+** | **+** | **-** | **-** | **-** | **-** |
| Sulfurous acid, cyclohexylmethyl octadecyl ester | **+** | **-** | **-** | **+** | **-** | **+** |
| Tricosane | **+** | **-** | **-** | **-** | **-** | **-** |
| 2-Methyl-7-phenylindole | **+** | **-** | **-** | **-** | **-** | **-** |
| Tetradecane, 4-ethyl- | **-** | **+** | **-** | **-** | **-** | **-** |
| Decane, 2,4,6-trimethyl- | **-** | **+** | **-** | **-** | **-** | **-** |
| Decane, 1,1'-oxybis- | **-** | **+** | **-** | **-** | **-** | **-** |
| Naphthalene, decahydro-2-methyl- | **-** | **+** | **-** | **-** | **-** | **-** |
| 2-Decen-1-ol, (E)- | **-** | **+** | **-** | **-** | **-** | **-** |
| Octane, 4-ethyl- | **-** | **+** | **-** | **-** | **-** | **-** |
| 4,4-Dipropylheptane | **-** | **+** | **+** | **-** | **-** | **-** |
| Tridecane, 1-iodo- | **-** | **+** | **+** | **-** | **-** | **-** |
| Undecane, 2-methyl- | **-** | **+** | **-** | **-** | **-** | **-** |
| 16-Octadecenal | **-** | **+** | **-** | **-** | **-** | **-** |
| 2,4,4,6,6,8,8-Heptamethyl-1-nonene | **-** | **+** | **-** | **-** | **-** | **-** |
| 17-Pentatriacontene | **-** | **+** | **-** | **-** | **-** | **-** |
| Triacontane | **-** | **+** | **-** | **-** | **-** | **-** |
| Octadecane, 2,2,4,15,17,17-hexamethyl-7,12-bis(3,5,5-trimethylhexyl)- | **-** | **+** | **-** | **-** | **-** | **-** |
| Oxalic acid, cyclohexylmethyl tetradecyl ester | **-** | **+** | **-** | **-** | **-** | **-** |
| Hexacosane | **-** | **+** | **-** | **-** | **-** | **-** |
| Cyclopentane, 1,1'-ethylidenebis- | **-** | **+** | **-** | **-** | **-** | **-** |
| Oxalic acid, allyl undecyl ester | **-** | **+** | **+** | **-** | **-** | **-** |
| Sulfurous acid, octadecyl 2-propyl ester | **-** | **+** | **+** | **-** | **-** | **-** |
| Thiophen-2-methylamine, N-(2-fluorophenyl)- | **-** | **+** | **+** | **-** | **-** | **-** |
| Cyclotrisiloxane, hexamethyl- | **-** | **+** | **-** | **-** | **-** | **-** |
| 2',4'-Dihydroxyacetophenone, bis(trimethylsilyl) ether | **-** | **-** | **+** | **-** | **-** | **-** |
| Eicosane, 9-octyl- | **-** | **-** | **+** | **-** | **+** | **-** |
| Silane, trichlorooctadecyl- | **-** | **-** | **+** | **-** | **-** | **-** |
| Sulfurous acid, dodecyl 2-propyl ester | **-** | **-** | **+** | **-** | **-** | **-** |
| Nonadecane | **-** | **-** | **+** | **-** | **-** | **-** |
| Decane, 2,4-dimethyl- | **-** | **-** | **+** | **-** | **-** | **-** |
| Oxirane, decyl- | **-** | **-** | **+** | **-** | **-** | **-** |
| 2,4,4,6,6,8,8-Heptamethyl-2-nonene | **-** | **-** | **+** | **-** | **-** | **-** |
| Carbonic acid, isobutyl octadecyl ester | **-** | **-** | **+** | **-** | **-** | **-** |
| 1-Octadecanesulphonyl chloride | **-** | **-** | **+** | **-** | **-** | **-** |
| Decane, 2,3,5-trimethyl- | **-** | **-** | **+** | **-** | **-** | **-** |
| Sulfurous acid, cyclohexylmethyl nonyl ester | **-** | **-** | **+** | **-** | **-** | **-** |
| Cyclopentanecarboxylic acid, 4-hexadecyl ester | **-** | **-** | **+** | **-** | **-** | **-** |
| Tetratetracontane | **-** | **-** | **+** | **-** | **-** | **-** |
| Decane | **-** | **-** | **-** | **+** | **-** | **-** |
| 3,5-Dimethyl-2-octanone | **-** | **-** | **-** | **+** | **-** | **-** |
| Decane, 2,3,5,8-tetramethyl- | **-** | **-** | **-** | **+** | **-** | **-** |
| Oxalic acid, cyclohexylmethyl undecyl ester | **-** | **-** | **-** | **+** | **-** | **-** |
| Methane, tricyclohexyl- | **-** | **-** | **-** | **+** | **-** | **-** |
| 2,6-Dimethyldecane | **-** | **-** | **-** | **-** | **+** | **-** |
| Decane, 3,7-dimethyl- | **-** | **-** | **-** | **-** | **+** | **-** |
| 10-Methylnonadecane | **-** | **-** | **-** | **-** | **+** | **-** |
| Decane, 2,3,7-trimethyl- | **-** | **-** | **-** | **-** | **+** | **-** |
| 2-Bromotetradecane | **-** | **-** | **-** | **-** | **+** | **-** |
| Sulfurous acid, cyclohexylmethyl tridecyl ester | **-** | **-** | **-** | **-** | **+** | **-** |
| Dodecane, 3-methyl- | **-** | **-** | **-** | **-** | **+** | **-** |
| Oxalic acid, cyclohexylmethyl dodecyl ester | **-** | **-** | **-** | **-** | **+** | **-** |
| 2-Nonadecanol | **-** | **-** | **-** | **-** | **+** | **-** |
| Undecane, 4,6-dimethyl- | **-** | **-** | **-** | **-** | **-** | **+** |
| Cyclohexasiloxane, dodecamethyl- | **-** | **-** | **-** | **-** | **-** | **+** |
| Oxazole, 2,4-dimethyl- | **-** | **-** | **-** | **-** | **-** | **+** |
| 2-Thiopheneethanol | **-** | **-** | **-** | **-** | **-** | **+** |

"+"means that the organic matter exists, and "-"means that the organic matter does not exist.


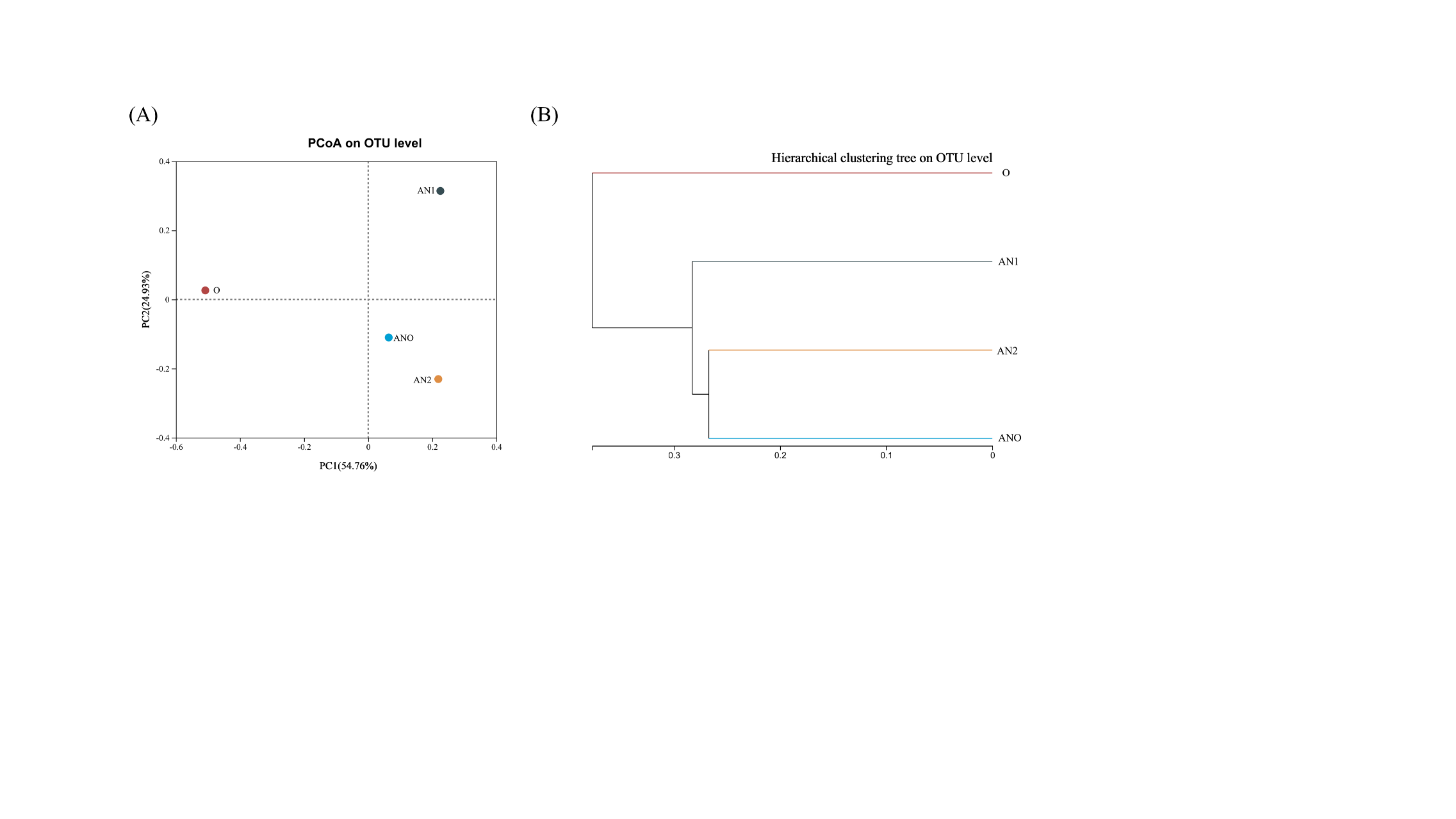


Figure S1 Beta diversity analysis (A) PCoA (B) OTU hierarchical cluster analysis
